# Scaffolds for Chondrogenic Cells Cultivation Prepared from Bacterial Cellulose with Relaxed Fibers Structure Induced Genetically

**DOI:** 10.3390/nano8121066

**Published:** 2018-12-17

**Authors:** Paulina Jacek, Marcin Szustak, Katarzyna Kubiak, Edyta Gendaszewska-Darmach, Karolina Ludwicka, Stanisław Bielecki

**Affiliations:** Institute of Technical Biochemistry, Lodz University of Technology, 4/10 Stefanowskiego Str., 90-924 Łódź, Poland; paulina.jacek@edu.p.lodz.pl (P.J.); marcin.szustak@edu.p.lodz.pl (M.S.); edyta.gendaszewska-darmach@p.lodz.pl (E.G.-D.); karolina.ludwicka@p.lodz.pl (K.L.); stanislaw.bielecki@p.lodz.pl (S.B.)

**Keywords:** bacterial nano-cellulose, 3D culture, scaffold, chondrogenic differentiation, *Komagataeibacter*, bacterial cellulose fiber structure

## Abstract

Development of three-dimensional scaffolds mimicking in vivo cells’ environment is an ongoing challenge for tissue engineering. Bacterial nano-cellulose (BNC) is a well-known biocompatible material with enormous water-holding capacity. However, a tight spatial organization of cellulose fibers limits cell ingrowth and restricts practical use of BNC-based scaffolds. The aim of this study was to address this issue avoiding any chemical treatment of natural nanomaterial. Genetic modifications of *Komagataeibacter hansenii* ATCC 23769 strain along with structural and mechanical properties characterization of obtained BNC membranes were conducted. Furthermore, the membranes were evaluated as scaffolds in in vitro assays to verify cells viability and glycosaminoglycan synthesis by chondrogenic ATDC5 cells line as well as RBL-2H3 mast cells degranulation. *K. hansenii* mutants with increased cell lengths and motility were shown to produce BNC membranes with increased pore sizes. Novel, BNC membranes with relaxed fiber structure revealed superior properties as scaffolds when compared to membranes produced by a wild-type strain. Obtained results confirm that a genetic modification of productive bacterial strain is a plausible way of adjustment of bacterial cellulose properties for tissue engineering applications without the employment of any chemical modifications.

## 1. Introduction

Mature cartilage has a very limited ability for repair, which has led to intense research toward the development of cell-seeded scaffolds. Preparation of an excellent scaffold material for cartilage tissue engineering is an ongoing challenge due to difficulty to acquire all of its desirable attributes simultaneously [1]. The most essential from these peculiar features are: fostering cell viability, chondrogenic differentiation, and extracellular matrix (ECM) production. Scaffolds provide a 3D surrounding for cells what prevents dedifferentiation of chondrocytes into a fibroblast-like cell type, what has been first shown for agarose gels [2]. Nevertheless, even slight changes in the structure of a material supporting differentiating chondrocytes can influence their fate, as recent research on electro-spun synthetic scaffolds indicated [3,4]. Furthermore, scaffolds must allow diffusion of nutrients and waste, therefore hydrogels are often used due to their high water content and hydrophilic nature [5]. Considering application in living organisms, evading response from the immunogenic system and integration with surrounding cartilage are of the highest importance [6,7]. Moreover, the material should provide mechanical support in order to accurately recreate the natural tissue endurance and to generate transportable products for clinical use [8]. Finally, a perfect scaffold material for cartilage regeneration should be moldable in order to fill the defect in natural tissue in vivo and, beforehand, to properly guide proliferating cells in vitro.

Many natural and synthetic materials have been tested as potential scaffolds in cartilage engineering, with best results obtained for polymers forming hydrogels, sponges or fibrous meshes [1,5,8,9]. Among others, natural substances are of the highest interest due to their abundance and general pro-chondrogenic properties [5]. One of the most frequently used natural hydrogels is agarose, consisting of polysaccharide. It has been shown to support chondrogenesis to higher extent when compared to other natural polymers including type I collagen, alginate, fibrin, and polyglycolic acid [10]. The presented study is focused on another polysaccharide-based biomaterial, namely cellulose produced by bacteria from *Komagataeibacter* genus (bacterial nano-cellulose, BNC).

Bacterial cellulose has been widely recognized as hydrogel-like material with well documented biocompatibility, resulting from its high purity (lack of hemicelluloses or lignin) [11,12]. BNC meets most of requirements for material supporting cartilage regeneration. Among others, bacterial nanocellulose features that predispose it for this application are: exceptionally high tensile strength, hydrogel-like properties (water constitutes at least 95% of its weight), and efficient bidirectional diffusion of water-soluble compounds [13,14]. Very recently, 3D organization of BNC multilayers together with the presence of surface cavities was shown to provide a natural biomechanical anchorage for cells and to promote collagen-I formation [15]. Biocompatibility of native BNC has already been confirmed profoundly. Numerous in vivo investigations have shown no foreign body reaction, no fibrous capsule or giant cells presence, and no development of inflammatory reaction both in short- and long-term implantations of BNC grafts [16,17,18]. It is worth mentioning that this form of cellulose is exceptionally moldable and multifarious implants have been obtained from BNC both in situ (during bacteria cultivation by various fermenter designs) and after biosynthesis by chemical and/or physical modifications [19,20,21,22,23]. Two of the most recent and intriguing achievements in this field are preparation of self-standing spheres with controllable sizes by conducting the fermentation process on hydrophobic surfaces [24] and obtaining a shape-memory membranes, self-arranging into tubes after mammalian cells printing [25]. Another example of preparation of BNC scaffold with controlled fibers structure (parallelly ordered) has been shown on NOC (nematic ordered cellulose) surfaces and proved to be efficient in supporting fibroblasts cultures in vitro [26,27]. The main potential obstacle in medical use of BNC as a material for implants is lack of cellulase activity in human body; therefore, it is widely assumed as non-degradable in vivo. Surprisingly, two in vivo studies conducted on rat models have recently reported resorption capability of bacterial cellulose. The first example was based on implementation of irradiated bacterial cellulose [28]. The second one describes preparation of bi-layered cell-free scaffolds from BNC-composites and its usage in parallel bone and cartilage regeneration [29]. Another approach aiming at assurance of faster BNC resorption after implementation in living organisms was the use of metabolically engineered *K. xylinus* strain for production of lysozyme-susceptible material. Obtained cellulose performed as effectively as native BNC when tested in vitro for supporting chondrogenesis of human mesenchymal stem cells [30]. Taking the advantage of cartilage regeneration potential of BNC, up to date, several research groups have tested this material in chondrocytes’ culturing with promising results. One of the first pieces of evidence of pro-chondrogenic properties of native and chemically modified BNC scaffolds, as distinct from 2D plastic support, was shown with bovine chondrocytes [31]. These results were followed by studies focused on introduction of micro-sized pores into nano-porous BNC, aiming at better ingrowth of chondrocytes. One of such examples was the preparation of cellulose by fermentation in media supplemented with paraffin droplets [32]. Even though sponge-like material obtained in this process was well-tolerated by human chondrocytes, infiltration of these cells into the scaffold was limited only to the most outside layers [32]. More recent results, obtained in mice with bilayer BNC composites (micro-porous layer prepared by freeze drying with alginate beads) implemented subcutaneously, confirmed good mechanical stability, maintaining structural integrity and supporting cell ingrowth of such composites [33]. Other composites of BNC with glycosaminoglycans (GAG) deposited on the surface of cellulose membrane were shown to increase chondrogenesis of native cellulose three-fold, measured *in vitro* by Alcian Blue staining (a well-established test for estimation of ECM production level) [29]. Most recently, BNC was shown to be crucial component of so-called nanofibrous microcarriers used for micro-tissue preparation under microgravity conditions, implemented in knee cartilage tissue regeneration *in vivo* in a rat model [34]. In the cited study microcapsules prepared with BNC noticeably better mimicked natural ECM when compared to chitosan-based ones (tested by GAG/DNA ratios and marker gene expression changes) [34]. Most of the progress in the field of development of chondrogenic cells supporting material based on bacterial cellulose included chemical or physical modifications, disturbing the natural form of BNC. Nevertheless, good integration of native bacterial cellulose membranes with mammalian cells has been shown constantly, with a recent example of rabbit bone marrow mesenchymal stem cells differentiating on this material into three lineages: chondrogenic, osteogenic, and adipogenic [35].

In the present study, BNC fibers structure has been changed without external disturbance of culturing process or application of any chemical modifications. Properties of BNC obtained from different *K. hansenii* or *K. xylinus* strains differ significantly in terms of membrane appearance (stiffness and consistency) and its morphology (fiber diameter and density of fiber network) [36,37]. Bacterial cellulose fiber is composed of numerous β-1,4-glucan chains continuously secreted from pores located along longitudinal axis of the bacterial cell, formed by terminal complexes of cellulose synthase subunits (TCS) [38,39,40]. In this work, genetically modified *K. hansenii* strain was designed and used for the production of BNC with increased porosity. Overexpression of target genes was achieved by the usage of previously described vector pTI99A invented and kindly shared by Kenji Tajima (Hokkaido University, Japan) [41]. Increased ability of modified bacterial cells to move on the surface of the medium and length of individual bacterial cells influenced BNC fiber organization. The phenotypic changes of modified bacterial strain resulted in relaxation of fibers structure visualized by means of electron scanning microscopy (SEM) and did not disturb cellulose chemical composition (verified by Fourier Transform Infrared (FTIR) spectra). However, increased porosity slightly influenced mechanical properties of the material when compared to cellulose produced by the parental strain. The second phase of presented study was devoted to evaluation of chondrogenesis supporting potential of obtained BNC scaffolds in in vitro tests. Our results clearly showed that BNC secreted by mutant strain preserved natural biocompatibility of BNC (verified by a mast cells degranulation test) and served as superior mammalian cells support both in terms of chondrogenic cells proliferation (measured by a resazurin-based Presto blue assay), morphology and extracellular matrix secretion (GAG production level estimated with Alcian blue staining) when compared to membranes produced by the wild-type strain. In accordance with our best knowledge, this is the first BNC scaffold in which improvement of pro-chondrogenic potential has been achieved solely by genetic modification of the bacterial strain.

## 2. Materials and Methods

### 2.1. Bacterial Strains, Mammalian Cell Lines, Culturing Media, and Conditions

*Komagataeibacter hansenii* Bacterial Strains

In this study *K. hansenii* ATCC 23769 (Hokkaido University, Japan) abbreviated WT and two its variants, obtained in this work (control—transformed with pTI99A vector, abbreviated Ctrl, and mutant—transformed with pTI99-motAB vector, abbreviated motAB+), were used. All *Komagataeibacter* strains were cultured in Hestrin-Schramm, HS, medium [42] with addition of 200 μg/mL ampicillin (AppliChem, Darmstadt, Germany) (abbreviated as HSA) and/or 1% (*v*/*v*) *Trichoderma reesei* cellulase (Sigma Aldrich, Saint Louis, MO, USA) (abbreviated as HSC), when needed. Inoculum for each experiment was freshly prepared as pre-culture (5 mL HS in tube) seeded by single colony from agar HS plate obtained by streaking from glycerol stocks preserved at −80 °C. Incubation at 30 °C in stationary or shaken (250 rpm) incubator lasted from 3 h to 168 h as indicated in detailed procedures’ descriptions below. Temperature and shaking conditions for all experiments were the same and are not mentioned below.

*Escherichia coli* TOP 10F strain

*E. coli* TOP 10F strain was used for DNA manipulation purposes only. Luria-Bertani (LB) medium [43], was used for competent cells preparation and vector propagation procedures. Super Optimal broth with Catabolite repression SOC medium [44], was used for regeneration after transformation. Recombinant selection was done on agar LB medium supplemented with 50 µg/mL ampicillin. Incubation at 37 °C in shaken (250 rpm) or stationary conditions lasted up to 24 h.

ATDC5 Mouse Chondrogenic Cell Line

The chondrogenic ATDC5 cell line, originating from mouse teratocarcinoma AT805 was purchased from the Health Protection Agency (supplied by Sigma Aldrich, Saint Louis, MO, USA). Cells were cultured in Dulbecco’s modified Eagle medium/Nutrient Mixture F12 (DMEM/F12 1:1, Thermo Fisher Scientific, Waltham, MA, USA) supplemented with 2 mM glutamine (Sigma Aldrich, Saint Louis, MO, USA), 5% Fetal Bovine Serum (FBS, Thermo Fisher Scientific, Waltham, MA, USA), 100 U/mL penicillin (Polfa Tarchomin, Warsaw, Poland), 100 mg/mL neomycin (Galfarm, Cracow, Poland), and 2.5 µg/mL amphotericin B (Thermo Fisher Scientific, Waltham, MA, USA). The ATDC5 cell line was incubated at 37 °C in a humidified atmosphere supplemented with 5% CO_2_.

RBL-2H3 Rat Basophilic Leukemia Cell Line

The rat basophilic leukemia RBL-2H3 cell line was purchased from Leibniz Institute DSMZ—German Collection of Microorganisms and Cell Cultures (Braunschweig, Germany). Cells were cultured in DMEM/RPMI 1640 (7:3) (Thermo Fisher Scientific, Waltham, MA, USA) supplemented with 10% FBS, 100 U/mL penicillin, 100 mg/mL streptomycin (Polfa Tarchomin, Warsaw, Poland) and 2.5 µg/mL amphotericin B. The RBL-2H cell line was incubated at 37 °C in a humidified atmosphere supplemented with 5% CO_2_.

### 2.2. DNA Manipulation Methods

Isolation of genomic DNA from *K. hansenii* ATCC 53582 and plasmid DNA from *E. coli* TOP 10F bacterial strains as well as DNA purification from agarose gel were performed by solid phase extraction on silica spin columns (Gene MATRIX Bacterial and Yeast Genomic DNA Purification Kit and GeneMatrix Basic DNA Purification Kit, EURx, Gdansk, Poland).

ColorTaq Polymerase (EURx, Gdansk, Poland) and C1000 Thermal Cycler PCR (Bio-Rad, Hercules, CA, USA) were used in all PCR amplification procedures (preparation of insert with *motAB* genes, control over pTI99-motAB vector preparation and verification of *K. hansenii* transformation) with appropriate primers (Table S1) synthesized at Genomed Ltd., Warsaw, Poland.

pTI99-motAB vector preparation was done with standard methods (restriction hydrolysis with BamHI and HindIII enzymes (FastDigest, Thermo Fisher Scientific, Waltham, MA, USA), overnight ligation catalyzed by T4 DNA ligase (Thermo Fisher Scientific, Waltham, MA, USA) and transformation by a heat shock method of competent *E. coli* TOP 10F cells (prepared in-house).

### 2.3. Transformation of K. hansenii ATCC 23769 Strain

Electro-competent *K. hansenii* ATCC 23769 cells were prepared from shaken culture in 50 mL of liquid HSC medium, which had reached optical density (λ = 600 nm) equal to 0.3–0.4. The cells were washed three times in 1 mM HEPES (Sigma Aldrich, Saint Louis, MO, USA) and frozen in 15% glycerol at −80 °C. Prior to transformation each 100 µL portion of frozen electro-competent *K. hansenii* cells was thawed on ice and transferred into 0.2 cm cuvettes. Electric pulse (2.5 kV, 5.9 ms) was generated by Gene Pulser (Bio-Rad, Hercules, CA, USA). Cell regeneration in HS medium lasted 3 h in shaking conditions. Recombinants selection was done on HSA agar plates after 96 h of incubation.

### 2.4. Bacterial Cells/Filaments Length Determination

Liquid cultures in HSC medium of three tested *K. hansenii* ATCC 23769 strain variants (wild-type, control, and mutant) were used for cells/filaments length determination. During six days of incubation with 24 h interval, 10 μL portions of cells suspension were fixed to the microscope slide and stained with crystal violet. From each slide 20 views were saved, with the use of light microscope Olympus BX 51 (Olympus, Tokyo, Japan) under 400 × magnification. Cells/filaments lengths were measured with Makroaufmassprogram software (https://ruedig.de/tmp/messprogramm.htm) for at least 10 cells chosen from each picture. Only well separated, single cells/filaments or, if cells/filaments were forming chains, only the ones with septa clearly visible were chosen for these measurements. Distribution of the obtained values comprised at least 120 individual measurements for each strain.

### 2.5. Bacterial Swarming Motility Assay

Pre-cultures of three tested *K. hansenii* ATCC 23769 strain variants (wild-type, control, and mutant) were diluted to reach optical density of 0.1 at 600 nm. Next, 2 μL portions from each equilibrated culture were inoculated onto five 0.3% agar HS plates containing 2% (*v*/*v*) cellulase. Spots of mutant culture were accompanied by spots of wild-type and control strains on the same plates. After each 24 h of the five-day incubation, the diameters of colonies were measured. 

### 2.6. Determination of Cellulose Density

Pre-cultures of three tested *K. hansenii* ATCC 23769 strain variants (wild-type, control and mutant) were used for inoculation of 45 mL SH or SHA medium in 250 mL flasks. Stationary cultures at 30 °C were continued for 4 days and BNC membranes were harvested, washed briefly in tap water and then purified with 0.1% NaOH overnight followed by several washes in distilled water until pH 7 was reached. The diameter of all obtained membranes was measured with 0.5 mm accuracy with a ruler and their thickness was estimated with 0.1 mm accuracy with an electronic caliper, in at least three places, after bending two times each membrane. Next membranes were completely dried at 80 °C in vacuum gel dryer (Model 543, Bio-Rad, Hercules, CA, USA). Dry mass of cellulose was measured on analytical balance. Cellulose density was calculated from Equation (1)

(1) $$D_{cel}=\frac{m_{dry\;cellulose}\;\left[g\right]}{V_{wet\;membrane}\;\left[m^{3}\right]}$$

mean values and standard deviations were obtained from four replicates.

### 2.7. Scanning Electron Microscopy of Bacterial Nanocellulose Membranes

For structural analysis BNC membranes obtained from 7 days-long cultures of *K. hansenii* ATCC 23769 and its variants, purified with use of SDS—method described elsewhere [45] were used. The membranes were freeze-dried in Christ Alpha model 1-4 LSC plus (Martin Christ Gefriertrocknungsanlagen GmbH, Osterode am Harz, Germany) and coated with gold. Scanning electron microscope FEI QUANTA 250 FEG (Thermo Fisher Scientific, Waltham, MA, USA), operating at 2 kV, was used for observation of three biological replicates at magnifications of 5000×, 20,000×, and 40,000×. Representative micrographs were taken in triplicates for each magnification. The diameters of cellulose fibers and pores were determined with the Makroaufmassprogram software, for 50 sites from each of the SEM micrographs (distribution of at least 150 results for each membrane were found). In this study, we use term ‘fiber’ for perceptible single filamentous structure seen in the SEM pictures (exemplary Figure S2 in supplementary material) without distinguishing microfibers, ribbons or bundles.

### 2.8. Bacterial Nanocellulose FTIR Characterization

BNC membranes after seven days of stationary cultivation in 50 mL liquid HS, were purified by single 0.1% NaOH rinsing (Chempur, Piekary Slaskie, Poland), and several deionized water washes, and then they were freeze-dried. The Spectrophotometer (Nicolet 6700, Thermo Fisher Scientific, Waltham, MA, USA) with the wave number range from 4500 to 600 cm^−1^ at a resolution of 0.5 cm^−1^ was used. Representative spectra from at least three independent measurements were analyzed.

### 2.9. Testing of Mechanical Parameters of BNC Membranes

BNC membranes after seven-day-long cultures (in rectangular bioreactors) of *K. hansenii* ATCC 23769 strain and its variants were purified with 0.1% NaOH and washed with water. Excess of water was removed by gentle squeezing on a fabric. Wet membranes were cut forming stripes of dimensions: 1.5 cm× 5.0 cm and tested for resistance to breaking. Tensile strength measurements were done with ZWICK/Roell testing machine (capacity up to 1 kN), with the parameters adjusted to 5 mm∙min^−1^ of clamps movement velocity and 15 mm of starting distance between the clamps. The tests were performed in air-conditioned room, at the temperature of 21 °C.

### 2.10. BNC Scaffolds Preparation and Mammalian Cells Seeding

The concentrations of 5% *v*/*v* of wild-type *K. hansenii* ATCC 23769 pre-culture and 10% *v*/*v* of control and mutant strains pre-cultures were used for inoculation of 1 mL of HS/HSA medium in 24-well culture plates. The same type of 24-well culture plates was used in mammalian cells tests. Incubation at 30 °C in stationary conditions was performed until the membranes reached approx. 1.5–2 mm thickness (in the case of wild type strain it was typically 2–3 days and in the case of control and mutant strains—5 days). Scaffolds were removed from 24-well culture plates, by putting a plastic inoculation loop under them, and soaked in 200 mL of 0.1% NaOH (ChemLand, Stargard Szczecinski, Poland), and cooled down at room temperature. Next, NaOH solution was replaced by distilled water (washes without squeezing the membranes). Afterwards scaffolds were rinsed with 200 mL of sterile miliQ water in 500 mL, screwed bottles, with daily water exchange, for 15 days. Additionally, thermal sterilization of the bottles with BNC membranes was repeated every five days. Each time the water was changed carefully by pouring the solution out of the bottles, leaving a little excess of water over the membranes, never rinsing or squeezing them (see Figure S4 for scaffolds appearance after this step). Finally, the scaffolds were placed into fresh 24 well plates by the use of plastic inoculation loop and flatted to unfold them on the bottoms of the wells. The plates were sealed in aluminum sachets and exposed on electron beam irradiation (radiation dose 25 kGy). Sterile BNC membranes were soaked in 1 mL of the appropriate complete medium (DMEM/F12 for ATDC5 line or DMEM/RPMI 1640 for RBL-2H3 cell line for at least 24 h at 37 °C. Then the medium was removed and cells were seeded onto BNC scaffolds at the same density of 1.5 × 10^5^ per well.

### 2.11. RBL-2H3 Mast Cell Degranulation

The analysis of mast cell degranulation was based on the measurement of N-acetyl-β-d-hexosaminidase (HEX) release from RBL-2H3 cells. After 24 h of incubation, the complete growth medium was replaced with a fresh one supplemented with dinitrophenyl (DNP)-specific mouse IgE antibody (0.5 µg/mL, Sigma Aldrich, Saint Louis, MO, USA) and RBL-2H3 mast cells growing on BNC scaffolds were incubated overnight. Then, cells were rinsed two times with 600 µl of PBS (Thermo Fisher Scientific, Waltham, MA, USA) and incubated for 20 min with DNP-BSA (10 ng/mL, Sigma Aldrich, Saint Louis, MO, USA). The reaction was stopped by placing the plates on ice. Subsequently, the plates were centrifuged at 300× g for 5 min at 4 °C and 60 µL of supernatant from each well was transferred into a fresh 96-well plate. The HEX enzymatic activity of the supernatants and cell lysates (after addition of 0.1% Triton X-100, Sigma Aldrich, Saint Louis, MO, USA) was determined using 8 mM p-nitrophenyl-N-acetyl-β-d-glucopyranoside (Sigma Aldrich, Saint Louis, MO, USA) in 0.08 M citric buffer (pH 4.5, prepared in-house) as a chromogenic substrate. The enzymatic reaction was stopped by addition 0.2 M glycine, pH 10.7 (Sigma Aldrich, Saint Louis, MO, USA). Triton X-100 was added to the wells used to determine the total cellular content of the HEX enzyme. Additional controls without antigen were used to measure spontaneous release. The experimental results were gathered by absorbance measurements at 405 vs. 492 nm using a Synergy 2 Microplate Reader (BioTek, Winooski, VT, USA).

### 2.12. Viability of Chondrogenic ATDC5 Cells

Cell viability was tested with a resazurin-based PrestoBlue assay (Thermo Fisher Scientific, Waltham, MA, USA). After 14 days of incubation with medium exchange every 2–3 days, BNC scaffolds with growing ATDC5 cells were transferred into clean 24-well plates. Next, 500 µL of DMEM/F12 medium and 40 µL of PrestoBlue cell viability reagent was added to each well and incubated for 90 min at 37 °C and 5% CO_2_. Fluorescent signal was captured by a Synergy 2 Microplate Reader at an excitation wavelength of 530 nm and an emission wavelength of 590 nm. Values of fluorescence magnitudes were used to calculate cell viability, expressed as a percentage of the viability of the cells growing on the surface of BNC produced by *K. hansenii* ATCC 23769 strain transformed with pTI99A core vector or with pTI99-motAB vector in comparison to viability relative to the growth of ATDC5 cells on WT BNC.

### 2.13. Morphology of Chondrogenic ATDC5 Cells

ATDC5 cells were incubated for up to 21 days and the medium was changed every 2–3 days. After first 24 h of incubation BNC scaffolds with attached cells were transferred into fresh plates. Morphology of cells was observed directly (without cells staining procedures) with a Leica M205 microscope (Leica Microsystems, Wetzlar, Germany) and images were captured on a Leica MC170 HD camera.

### 2.14. Production of Glycosaminoglycans by Chondrogenic ATDC5 Cells

Glycosaminoglycans synthesis was evaluated by staining the cell layers with Alcian Blue 8GX (Sigma Aldrich, Saint Louis, MO, USA). After 14 days of incubation with medium exchange every 2–3 days, BNC scaffolds with growing ATDC5 cells were transferred to clean 24-well plates. Cells were rinsed three times with PBS and fixed with 5.0% formaldehyde solution (Chempur, Piekary Slaskie, Poland) for 10 min. Subsequently, the cells were stained with 0.1% Alcian Blue in 0.1 M HCl for 24 h, at room temperature and rinsed three times with PBS solution. Alcian Blue-stained cultures were extracted with 6 M guanidine-HCl for 24 h at room temperature and the absorbance was determined at 610 nm by Synergy 2 Microplate Reader. In order to compare the samples between each other absorbance was divided by cells viability (Equation (2)).

(2) $$\%GAG=\;\frac{\frac{sample\;absorbance-bacground\;absorbance}{sample\;viability}}{\frac{control\;absorbance-background\;absorbance}{control\;viability}}\;\times 100\%$$

### 2.15. Statistical Analysis

All data except from bacterial cells’ lengths and cellulose fibers and pore dimensions are presented as the mean ± SD (*N* ≥ 3). Significance analysis for measurements of dimensions of bacterial colonies on soft agar plates, as well as cellulose density and biomechanical outcomes were done with *t*-test. Mammalian cells viability assay and level of glycosaminoglycans were analyzed by one-way ANOVA with a Bonferroni test. To analyze significance differences in mast cell degranulation, two-way ANOVA with Dunnett test was used. In all experiments, differences between groups were rated significant at a probability error *p* < 0.05.

## 3. Results

### 3.1. Preparation and Phenotype Characterization of K. hansenii ATCC 23769 motAB+ Strain

For this study a non-motile, *K. hansenii* ATCC 23769 strain was chosen [40]. The cellulose membranes produced by this strain are more gel-like when compared to other effective BNC producers, e.g., *K. hansenii* ATCC 53582. Furthermore, the target strain is relatively easily transformable when compared to *K. hansenii* ATCC 53582 strain and some *K. xylinus* strains (data not shown). Vector used for induction of *motA* and *motB* genes over-expression was prepared from the published previously pTI99A vector [41] and was obtained by the means of standard methods. Transformation of *K. hansenii* cells was conducted via electroporation and monitored by colony PCR (Figure S1). Wild type strain did not show ampicillin resistance, therefore recombinant selection was straightforward.

Basic bioinformatics analysis predicted that the proteins encoded by *motA* and *motB* genes belonged to the superfamily of proteins forming proton pumps with documented roles in bacterial cell motility (MotA, MotB) [42,43], cellular division (TolQ, TolR) [44,45] and active transport (ExbB, ExbD) [46,47,48,49]. Even though *K. hansenii* MotA and MotB proteins share the highest sequence similarity with motor stator proteins, essential for flagellum rotation in numerous bacterial species, their exact role in tested, non-flagellated *K. hansenii* ATCC 23769 strain is unclear. It is probable that the torque produced by this proton pump is used in other, unknown yet motility mechanism. On the other hand, its involvement in cell division and/or transport cannot be excluded. It was assumed that both induction of motility and/or disturbance in cells divisions, resulting in cells elongation or filamentation, should influence the spatial organization of fibers in cellulose membranes. This assumption was based on well known fact of secretion of β-1,4-glucan chain form TCS’s placed along longitudinal axis of the bacterial cell [34]. Therefore, bacterial strain phenotype characterization was limited to cells/filaments sizes and motility estimation.

**Figure 1:**
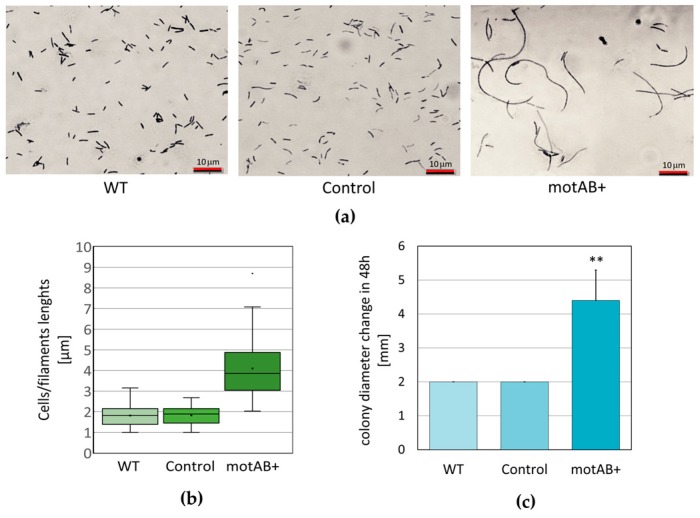
Phenotypes of *K. hansenii* ATCC 23769 strain variants (WT = wild-type *K. hansenii* ATCC 23769 strain, Ctrl = strain transformed with pTI99A core vector; motAB+ = mutant transformed with pTI99-motAB vector). (**a**) Bacterial cells/filaments length estimation—representative microphotographs from light microscope Olympus BX 51 of fixed bacterial cells/filaments, stained with crystal violet; (**b**) Bacterial cells/filaments length estimation—box plot showing distributions of 120 measurements of bacterial cells/filaments lengths for each strain; (**c**) Colony spreading assay (swarming motility on soft agar) differences in colonies’ diameters from fifth and third day of incubation in mm are shown. Mean values were calculated from five biological replicates, error bars represent standard deviations, statistical significance was estimated with *t*-test; ** denotes *p* < 0.005.

Microscopic evaluation of fixed cells suspensions from six days of incubation in static culture has undoubtedly shown that overexpression of *motAB* genes resulted in prominent elongation of cells, tending to gather in long chains or to form filaments (Figure 1a,b). Using crystal violet staining, we were unable to clearly visualize septa in all the observed cellular structures and therefore, elucidate if the cells are dividing and forming chains or rather tend to become filaments. Nevertheless, clear difference in the mutant strain phenotype when compared to the wild-type and control strains is evident.

Next, soft-agar motility assay was performed in order to examine swarming motility of bacteria [50,51,52]. Briefly, density-equilibrated cultures of tested strains were spotted on a semi-solid agar medium and spreading of ‘colonies’ was measured every day in millimeters (mm). Diameters of spots made on agar plates begun to spread on the third day of incubation, but in the case of wild type and control strain they widen only by 2 mm during the next 48 h, while spreading of motAB+ strain was twice as effective (Figure 1c).

### 3.2. Morphology and Mechanical Properties of BNC Membranes Produced by K. hansenii ATCC 23769 Strain Variants

Genetically modified strain was able to produce BNC membranes, which macroscopic appearance was more gel-like and loosely structured than the material obtained from parental *K. hansenii* ATCC 23769 strain (manual judgment). These observations were verified first by the measurement of cellulose density in the obtained membranes. Mutant strain produced membranes with approximately 0.20 g/cm^3^ less cellulose when compared to both control and parental strain in the same culturing conditions. Namely, density of cellulose (estimated as dry weight of cellulose divided by volume of the wet, never compressed membrane) was 1.25 ± 0.12 g/cm^3^ and 1.23 ± 0.10 g/cm^3^ for wild type and control strains accordingly and 1.03 ± 0.09 g/cm^3^ for motAB+ strain. The statistical significance of differences observed between mutant and both control and WT-strain-produced membranes was estimated by *t*-test and showed *p* < 0.05.

**Figure 2:**
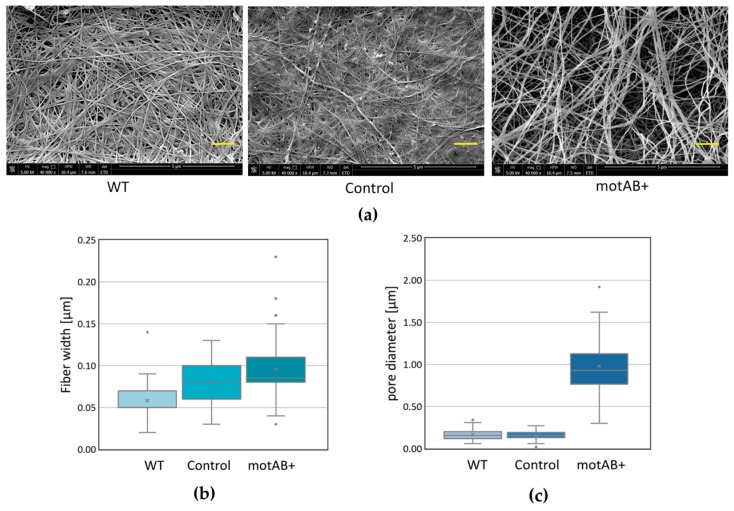
Morphology changes in BNC membranes produced by *K. hansenii* ATCC 23769 strain variants (WT BNC = wild type strain, Ctrl BNC = strain transformed with pTI99A core vector; motAB+ BNC= mutant transformed with pTI99-motAB vector). (**a**) Representative SEM microphotographs (40,000× magnification). Yellow scale bars correspond to 1 µm; Distribution of (**b**) fiber width sizes; (**c**) Distances between fibers measured for 50 sites from three representative microphotographs each.

These macroscopic results were further confirmed by scanning electron microscopy analysis, which revealed clearly more spacious cellulose fibers organization in motAB+ strain BNC when compared to both wild-type and control strain derived BNCs (Figure 2a). Moreover, the fibers were widen (Figure 2a,b). An increase of pore sizes, estimated from SEM pictures (Figure 2c) was the most profound feature of motAB+ BNC. By means of Fourier transform infrared spectroscopy (FTIR) it was confirmed that no new functional groups had been introduced in mutant-derived BNC as no changes in peaks at wavenumber range of 1800–1400 cm^−1^ was detected when compared to both wild-type and control membranes (Figure S3) [53].

**Figure 3:**
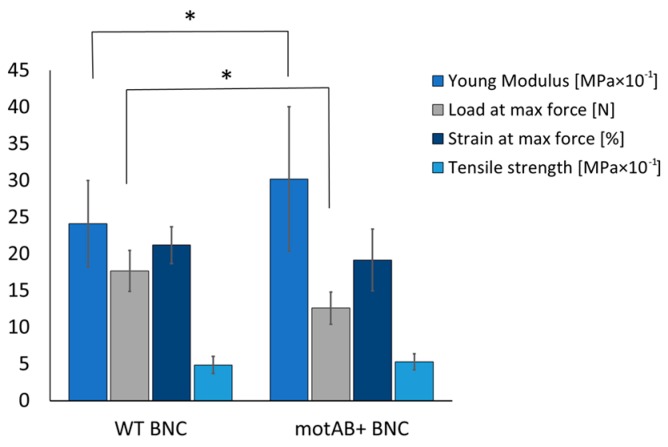
Mechanical parameters of native (WT BNC) and mutant–derived (motAB+ BNC) bacterial cellulose membranes. Significance calculated with *t*-test; * denotes *p* < 0.05.

Characterization of the obtained material was supplemented with testing for resistance to stretching. Mutant-derived membranes were more prone to breakage (load at maximum force was lowered by 28%) when compared to the wild type derived membranes (Figure 3). Such an observation can be related to more relaxed fiber structure as bacterial cellulose membranes with increased porosity have been reported as more prone to mechanical distortion than native ones [46,47]. At the same time a small Young’s modulus may suggest higher elasticity of cellulose samples obtained from motAB+ strain, what may relate to an increased fiber width. The other parameters did not change significantly, however elongation of the mutant derived membranes seems to be lower, what correlates with lower force needed for its breakage.

### 3.3. Biocompatibility and Pro-Chondrogenic Properties of BNC Membranes Produced by K. hansenii ATCC 23769 Strain Variants

**Figure 4:**
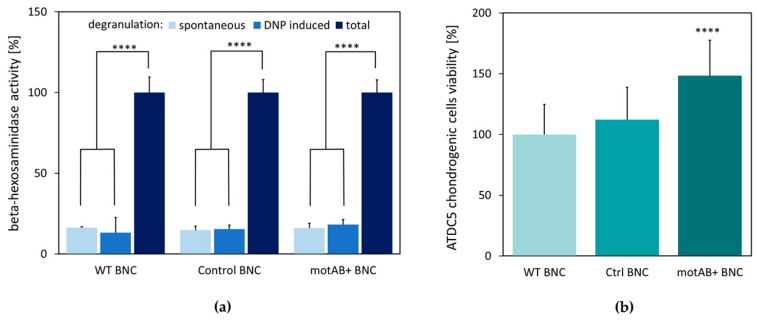
Verification of modified BNC material safety in in vitro experiments. (**a**) Measurement of mast cells degranulation with β-hexosaminidase activity assay. Dark blue—a positive control—cells treated with Triton X-100; blue—degranulation induced by addition of DNP, pale blue—spontaneous degranulation observed with no additives Statistics: **** denotes *p* < 0.0001 when compared to positive control; (**b**) Viability of ATDC5 cells growing on mutant-derived BNC material vs cells cultured on unmodified BNC (WT), whose viability was assumed to be 100% measured by PrestoBlue assay after 14 days of incubation. Data represent the means ± SD; Statistics: **** denotes *p* < 0.0001 when compared to wild type control.

Increased porosity of the new BNC material was a fundamental trigger for subsequent in vitro testing of its potential in tissue engineering applications. For these purposes BNC scaffolds were prepared in 24-well plates, what assured formation of membranes accurately shaped for cellular assays. We have conducted preliminary in vitro studies by direct seeding of the mammalian cells on the surface of all prepared BNC scaffolds to have insight into the behavior of the obtained material in a biological environment. As a result, the potential increased immunogenicity of motAB+ BNC material was excluded by mast cells degranulation assay with N-acetyl-β-d-hexosaminidase release. We have applied this method previously in the study of bacterial nanocellulose composites with polypropylene mesh [48]. All tested BNC scaffolds showed the same, low level of mast cells degranulation. Importantly, the effect observed for motAB+ BNC was not elevated when compared to starting material (BNC derived from wild-type bacterial strain) (Figure 4a), indicating similar non-immunostimulatory properties for both, native and mutant-derived BNC.

Another important feature of a biomaterial tested as the potential scaffold is its ability to support cells proliferation. Chondrogenic cells (ATDC5 cell line) viability was tested on three BNC scaffolds by a resazurin-based PrestoBlue assay. A nearly 50 percent increase in viable cells number was observed on membranes with relaxed fiber structure when compared to both, wild-type and control membranes (Figure 4b).

**Figure 5:**
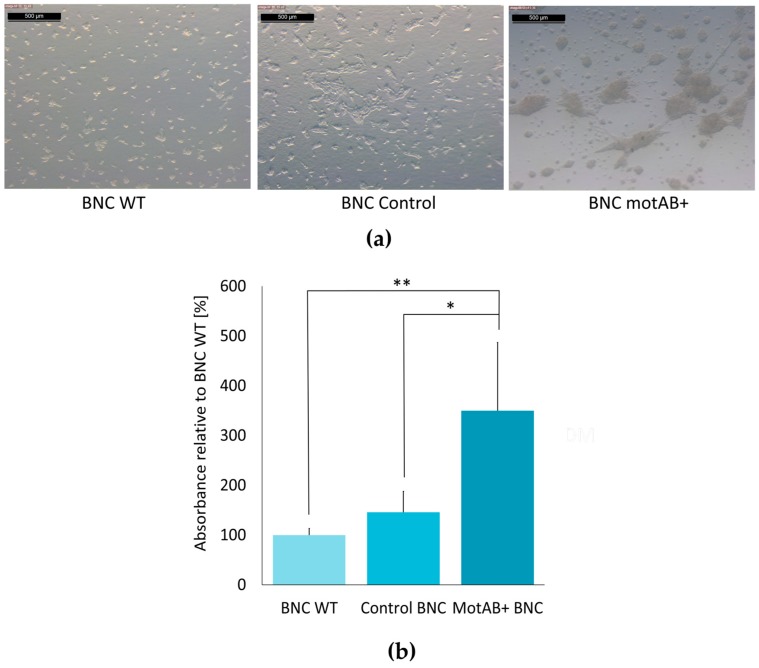
Chondrogenic-like behavior of ATDC5 cells cultured on BNC scaffolds produced by: wild type *K. hansenii* ATCC 23769 strain (WT BNC); control *K. hansenii* ATCC 23769 transformed with pTI99A (Ctrl BNC); mutant *K. hansenii* ATCC 23769 motAB+ strain (motAB+ BNC). (**a**) Morphology of ATDC5 cells observed under light M205 microscope, equipped with a Leica MC170 HD camera. Representative images (100× magnification) from three-week long culture are shown. Scale bars correspond to 500 µm; (**b**) Levels of glycosaminoglycan synthesis in Alcian blue stained ATDC5 cells. Bars on the graph represent levels of sample absorbance relative to absorbance of control cells seeded on BNC WT. Results obtained for BNC seeded cell corrected by subtraction of no-cell scaffold absorbance; Statistics: ** denotes *p* < 0.001; * denotes *p* < 0.05

Having confirmed preservation of non-toxic and non-immunostimulatory features of BNC, chondrogenic-like behavior of ATDC5 cell line was monitored as well. Cell morphology was evaluated during three-week long cultures on all types of BNC scaffolds (Figure 5a). Contrary to the cells seeded on 2D plastic support of the standard culturing plate in both growth and differentiating medium (see Figure S5), ATDC5 cells seeded on all BNC scaffolds tended to group into characteristic nodules. In the microphotographs presented in the Figure 5a it may be noticed that the cells with prolonged, fibroblast-like morphology hardly could be found on any of cellulosic materials. Furthermore, mutant-derived BNC seemed to give the best outcome out of all three scaffolds since the nodules were separated to the highest extent and contained more cells (see also Figure S6 for representative pictures after Alcian blue staining). Both these results (rounded morphology of the cells and their growth in nodules) are first lines of evidence used for confirmation of chondrogenic-like behavior of cells [1].

The presence of glycosaminoglycans (GAG)—extracellular matrix (ECM) components secreted by the cells is most frequently used as quantitative test for chondrogenic differentiation initiation [9]. Alcian blue binding assay was conducted in order to compare ECM production in the cells seeded on the three BNC supports obtained in this study (namely: bacterial nanocellulose produced by wild-type, control and mutant *K. hansenii* ATCC 23769 strains). Interestingly, ATDC5 cells seeded on motAB+ BNC appeared to secrete the highest levels of GAGs out of all BNC scaffolds tested (Figure 5b).

## 4. Discussion

The phenotype of *K. hansenii* ATCC 23769 motAB+ mutant strain was clearly different from a wild-type one. We observed significant bacterial cells elongation (or formation of filaments) and intensification of colony spreading ability (Figure 1). Simultaneously, the phenotype of the control strain (*K. hansenii* ATCC 23769 transformed with empty pTI99A vector) was unchanged. Therefore, it can be concluded that the observed phenotypic changes of mutant motAB+ strain were induced by the genetic change (overexpression of the two genes: *motA* and *motB*) not by an introduction of an additional plasmid. In the case of very long cellular structures (as the ones shown in Figure 1a, right panel) we found it difficult to elucidate whether we observe formation of chains by divided cells or formation of filaments (not fully divided structures). Even though measuring only the lengths of well separated cells (single or in chains with distinguishable septa), the differences, when compared to wild-type and control strains, are significant (Figure 1b). Bacterial cell elongation is commonly interpreted as the disturbance of cell division process. It may occur as a result of change in the cell cycle control or at the stage of daughter cell’s disintegration (outer membrane dissociation) process. Similar phenotype was observed in *E. coli* mutant strains and was interpreted as an evidence for engagement of TolA and Pal proteins in outer membrane invagination process [49]. In *E. coli* TolA-Pal complex is energized by TolQ TolR proteins forming a proton pump [50]. Genes used for overexpression in mutant strain tested in this study share only moderate sequence similarity with TolQ TolR proteins (based on blast searches). Therefore, mutant cells elongation (or filament formation) observed in this work may be a premise of involvement of the *motAB* genes in outer membrane invagination in *K. hansenii* species. On the other hand, yet there is not enough experimental data supporting this hypothesis gathered. Spreading of the bacterial colonies on the soft agar is generally interpreted as presence of swarming motility in the tested bacterial strains [51,52,53]. In the presented study stimulation of colony spreading in *K. hansenii* ATCC 23769 motAB+ mutant strain in contrast to both wild-type and control strains was observed (Figure 1c). No molecular mechanism responsible for motility has been described in *K. hansenii* ATCC 23769 up to date and no flagellum or pili structural genes have been identified in its’ genome sequence (data not shown). Therefore, it is not possible to speculate whether colony spreading effect is due to increased energizing of some yet-unknown motility machinery by additional copies of *motAmotB* genes products or if it is correlated with cell division process as well. In order to elucidate the exact role of *motAmotB* genes in *K. hansenii*, more genetic studies are needed.

Nevertheless, the phenotype described above was found to be interesting due to its potential influence on fiber structure and, in consequence, on morphology of the BNC membranes produced by the mutant strain. It is a well-known fact that bacterial cellulose fiber is composed of microfibrills, composed of nanofibrills formed by β-1,4-glucan chains secreted, from several TCSs (terminal complexes of cellulose synthase) placed along longitudinal axis of the bacterial cell [38,39]. Moreover, bacterial cell division event is most probably reflected as branching in cellulose fibers, since they are continuously secreted by parental and daughter cells [40,54]. Furthermore, it has been shown, by observation of the fiber secretion on the surfaces of controlled structure, that fibers reflect the track of bacterial cell movement [55]. Finally, the same authors demonstrated that wider fibers originate from longer cells (average cells sizes were 10 µm and 2 µm for *K. hansenii* ATCC 53582 and *K. hansenii* ATCC 23769 strains, accordingly) [55]. In this presented study BNC membranes produced by elongated, mutant *K. hansenii* ATCC 23769 cells were tested by SEM and compared to both wild-type and control strains-derived material (Figure 2). Being aware that SEM images analysis is not a perfect method for fiber width estimation, due to gold deposition, fiber width measurements were conducted only in order to compare the distributions of sizes between BNC samples, not to determine the objective fiber sizes. Widening of the mutant-derived cellulose fibers is noticeable from the data gathered in this study (Figure 2) but is not definite, what needs further, more exact elucidation to be properly compared with the previous research, mentioned above [55]. Nonetheless, already at this stage of analysis, we could observe a logical correlation between cells/filaments and fibers sizes, i.e., mutant strain possessed twice longer cells than a wild-type one (on average ~3.9 µm and ~1.8 µm, respectively) (Figure 1b), what was reflected in fiber diameter increase of roughly 300 nm (Figure 2b). What is much more clearly pronounced in the obtained results is the enlargement of pores in mutant-derived BNC membranes (Figure 2c). When motAB+ sample is compared to the wild-type, fiber network seems to be much denser in the latter one. These effects may be caused by cell division impairment resulting in sporadic events of cells dissociation and formation of fewer branches in cellulose fibers [54]. Moreover, we cannot exclude that relaxed fiber architecture is due to the change in motAB+ strain motility (Figure 1c), in analogy to the previous results demonstrating correlation between bacterial cell movement and cellulose fiber deposition [55,56].

Relaxed fiber structure in mutant-derived BNC membranes, revealed in SEM, was also confirmed by measurements of cellulose density and mechanical response of the obtained material (Figure 3). Dry weight of cellulose measurement in membranes produced in static cultures of the three strains showed decrease of its content by 0.23 ± 0.15 g/cm^3^ and 0.21 ± 0.14 g/cm^3^ of mutant-derived material in relation to wild-type and control strain-derived, accordingly. Such a result has its direct impact on mechanical performance of the material (Figure 3). Lower density of membranes produced by mutant strain caused a slight reduction of its resistance to stretching, evidenced by lowering of load at maximum force. However, an increase of Young’s modulus, which is a kind of stiffness indicator, stands in opposition to previously published studies on bacterial cellulose with increased porosity [46]. More plastic behavior of mutant derived samples may result from altered cellulose fibers width that was increased in motAB+ group (Figure 2). Even though mutant-derived material is more porous, i.e., more prone to damage, the natural good mechanical characteristics of BNC are preserved as the differences do not exceed 30% of the initial values (namely 25% increase in Young modulus and 28% decrease of load at maximum force). Finally, no disturbance in chemical groups present in mutant-derived BNC material was detected by means of FTIR spectroscopy (Figure S3), therefore all changes in the membranes’ morphology and mechanical features are solely dependent on fiber architecture rearrangement. Summarizing material characterization tests, it can be concluded that genetic modification of the bacterial strain resulted in increased pore sizes, influenced mechanical response of the material and did not disturb chemical structure of the bacterial cellulose.

The motAB+ BNC was further examined as a potentially interesting material supporting cartilage regeneration. Purification method with NaOH has been chosen for fabrication of scaffolds because it was previously shown to be an efficient method in removal of bacterial cells [57]. Following steps of washes were conducted in high excess of water, prolonged to two-weeks’ time and disturbed by three thermal sterilization procedures in order to ensure the removal of bacterial cells’ debris. Finally, the scaffolds have been radially sterilized, which could enhance its resorption capability as it was shown in vivo by others [28]. With the advent of many biomaterial compositions, it has become the prime focus of this field to ensure safety of the newly developed material. Taking advantage of the fact that BNC is already well-established as a biocompatible material the wild-type derived native cellulose was used as a positive control in all mammalian cells experiments [18].

Mast cell degranulation is often used as an in vitro test reflecting the potential of the tested biomaterial to induce acute and/or chronic inflammatory host organism responses, which are particularly triggered by macrophages, T-cells, and mast cells [7]. Essentially, the recruitment of inflammatory cells to the site of implantation of foreign material has been ascribed to mast cells belonging to the population of leukocytes [58]. Once activated, mast cells secrete numerous vasoactive and pro-inflammatory mediators, stored in secretory granules [59]. Those agents, critical in progression of inflammatory response, are released in degranulation process induced by both immunoglobulin E (IgE) and non-immunologic substances. These processes have been shown to play a major role in the inflammatory response to biomaterials [60]. Structurally modified cellulose obtained in the present study has shown same level of spontaneous mast cells degranulation as native, wild-type BNC (Figure 4). Therefore, newly obtained material should preserve non-immunostimulatory properties of native BNC.

The proper cell–scaffold interaction is crucial for a good scaffold material serving as a support in tissue engineering, what is manifested by higher cells viability [1,8]. Interestingly, motAB+ BNC appeared to support ATDC5 cells growth to higher extent than the wild-type BNC scaffold (Figure 4b). This observation might be explained by the adherence of ATDC5 cells to the mutant-derived material, due to relaxed BNC fibers structure, but further experimental evidence is needed to verify this hypothesis. Stimulation of chondritic cells proliferation is an important advantage of the motAB+ BNC material for potential future application in tissue engineering [8].

Cell morphology analysis and evaluation of extracellular matrix secretion is of prime importance in qualification of a new material as a support for chondrogenic cells culturing [10]. The long expansion time and multiple passaging often lead to ‘dedifferentiation’ of chondrocytes. These cells cultivated in vitro on the plastic surfaces dedifferentiate quickly to less specific cells with enhanced ability to proliferate. Morphological changes following chondroneogenesis pathway can be clearly distinguished as cells tend to form nodules (groups of cells connected with each other) and are rounded in shape (in contrast to stretched, elongated fibrous-like cells) [35]. The second approach to verify and quantify the advancement of chondrogenic differentiation is estimation of glycosaminoglycans (an important ECM component) synthesis level, most commonly by staining with Alcian blue [10]. In the present research the evidences for chondrogenic-like behavior of ATDC5 cells seeded on BNC scaffolds were delivered by the results of both tests. As it can be clearly seen in the micro-photographs in Figure 5a, and supplementary Figure S6, ATDC5 cells seeded on all three BNC scaffolds exhibited chondrogenic morphology, manifested most profoundly on motAB+ BNC support. The results of GAG secretion estimation indicated three times higher level in ATDC5 cells cultured on the motAB+ BNC when compared to WT BNC, further reaffirming an increase of pro-chondrogenic properties of mutant-derived BNC (Figure 5b). This observation may be explained by easier contact between cells and cellulose fibers in modified material. The presence of cellulose fibers in scaffold material has recently been proven by Wang et al., 2018 to be crucial in mimicking ECM environment [34]. In the cited research microcapsules prepared with and without BNC fibers addition were used for the propagation of microtissue from bone-marrow derived mesenchymal stem cells under microgravity conditions in non-differentiating medium. Chondrogenic-like morphology and increased GAG-secretion levels were evident for BNC-containing microcarriers as compared to the ones without BNC. Microtissues and microcarriers without seeded mesenchymal cells were used in microsurgery tests and revealed great potential in the regeneration of knee cartilage in rats [34]. The results indicating an induction of changes in chondrogenesis by means of subtle structural changes of the scaffold supporting cells differentiation have been published before with other materials as well [3,4]. In the both cited studies, the electrospun nano-sized fibers were less supportive for chondrogenesis than micro-sized ones. Importantly, even though many reports indicated the stimulation of ECM secretion from chondrocytes by BNC scaffolds both in vitro and in vivo [29,30,31,32,33,34,35], our material is the first one modified by means of genetic engineering and showing an increasing GAG secretion when compared to the native BNC (Figure 5b). The more so because most advances in the BNC usage as scaffold material in tissue engineering has been achieved by chemical and physical modifications, thus bringing the risk of impairing natural biocompatibility properties of BNC [11,12,20,21,29,32,33,34].

## 5. Conclusions

In the present study, new BNC material with relaxed fiber structure was obtained by means of genetic engineering of *K. hansenii* ATCC 23769 strain. The mutant-derived BNC appeared to be very promising as a support for chondrogenic cells propagation and promoted their chondrogenic-like behavior. Simultaneously the material was proved to preserve biocompatibility and chemical properties of BNC derived from parental strain. Therefore, it could be further used as starting material for physical or chemical modification in order to develop new, improved composites, which should greatly extend its potential applications in regenerative medicine.

## Supplementary Materials

The following are available online at http://www.mdpi.com/2079-4991/8/12/1066/s1, Figure S1: Confirmation of *K. hansenii* ATCC 23769 strain transformation by colony PCR, Figure S2: Example of fiber width and pore sizes estimation Figure S3: FTIR spectra collected for freeze-dried membranes produced by *K. hansenii* ATCC 23769 wild type strain and its variants: control (transformed with pTI99A vector) and mutant (motAB+ strain), Figure S4. Macroscopic appearance of BNC scaffolds used in the study. Figure S5: Morphology of ATDC5 cell line grown on 2D support Table S1: Sequences of DNA oligonucleotides used in PCR. Figure S6. Morphology of ATDC5 cell line grown on BNC scaffolds, stained with Alcian blue.

## References

[1] Foster N.C., Henstock J.R., Reinwald Y., El Haj A.J. (2015). Dynamic 3D culture: Models of chondrogenesis and endochondral ossification. Birth Defects Res. Part C Embryo Today Rev..

[2] Benya P.D., Shaffer J.D. (1982). Dedifferentiated chondrocytes reexpress the differentiated collagen phenotype when cultured in agarose gels. Cell.

[3] Chen H., Huang X., Zhang M., Damanik F., Baker M.B., Leferink A., Yuan H., Truckenmüller R., van Blitterswijk C., Moroni L. (2017). Tailoring surface nanoroughness of electrospun scaffolds for skeletal tissue engineering. Acta Biomater..

[4] Stenhamre H., Thorvaldsson A., Enochson L., Walkenström P., Lindahl A., Brittberg M., Gatenholm P. (2013). Nanosized fibers’ effect on adult human articular chondrocytes behavior. Mater. Sci. Eng. C.

[5] Kim I.L., Mauck R.L., Burdick J.A. (2011). Hydrogel design for cartilage tissue engineering: A case study with hyaluronic acid. Biomaterials.

[6] Anderson J.M., Rodriguez A., Chang D.T. (2008). Foreign body reaction to biomaterials. Semin. Immunol..

[7] Tang L., Jennings T.A., Eaton J.W. (1998). Mast cells mediate acute inflammatory responses to implanted biomaterials. Proc. Natl. Acad. Sci. USA.

[8] Spiller K.L., Maher S.A., Lowman A.M. (2011). Hydrogels for the Repair of Articular Cartilage Defects. Tissue Eng. Part B Rev..

[9] Kudva A.K., Luyten F.P., Patterson J. (2017). Initiating human articular chondrocyte re-differentiation in a 3D system after 2D expansion. J. Mater. Sci. Mater. Med..

[10] Mouw J.K., Case N.D., Guldberg R.E., Plaas A.H.K., Levenston M.E. (2005). Variations in matrix composition and GAG fine structure among scaffolds for cartilage tissue engineering. Osteoarthr. Cartil..

[11] Ludwicka K., Jedrzejczak-Krzepkowska M., Kubiak K., Kolodziejczyk M., Pankiewicz T. (2016). Medical and Cosmetic Applications of Bacterial NanoCellulose. Bact. Nanocellulose.

[12] Moniri M., Boroumand Moghaddam A., Azizi S., Abdul Rahim R., Bin Ariff A., Zuhainis Saad W., Navaderi M., Mohamad R. (2017). Production and Status of Bacterial Cellulose in Biomedical Engineering. Nanomaterials.

[13] Lee C.M., Gu J., Kafle K., Catchmark J., Kim S.H. (2015). Cellulose produced by Gluconacetobacter xylinus strains ATCC 53524 and ATCC 23768: Pellicle formation, post-synthesis aggregation and fiber density. Carbohydr. Polym..

[14] Bielecki S., Kalinowska H., Krystynowicz A., Kubiak K., Kodziejczyk M., de Groeve M. (2012). Wound Dressings and Cosmetic Materials from Bacterial Nanocellulose. Bact. Nanocellulose.

[15] Vielreicher M., Kralisch D., Völkl S., Sternal F., Arkudas A., Friedrich O. (2018). Bacterial nanocellulose stimulates mesenchymal stem cell expansion and formation of stable collagen-I networks as a novel biomaterial in tissue engineering. Sci. Rep..

[16] Andrade F.K., Silva J.P., Carvalho M., Castanheira E.M.S., Soares R., Gama M. (2011). Studies on the hemocompatibility of bacterial cellulose. J. Biomed. Mater. Res. Part A.

[17] Grobelski B., Wach R.A., Adamus A., Olejnik A.K., Kowalska-Ludwicka K., Kolodziejczyk M., Bielecki S., Rosiak J.M., Pasieka Z. (2014). Biocompatibility of Modified Bionanocellulose and Porous Poly(ϵ-caprolactone) Biomaterials. Int. J. Polym. Mater. Polym. Biomater..

[18] Pértile R.A.N., Moreira S., Gil da Costa R.M., Correia A., Guãrdao L., Gartner F., Vilanova M., Gama M. (2012). Bacterial Cellulose: Long-Term Biocompatibility Studies. J. Biomater. Sci. Polym. Ed..

[19] Kowalska-Ludwicka K., Cala J., Grobelski B., Sygut D., Jesionek-Kupnicka D., Kolodziejczyk M., Bielecki S., Pasieka Z. (2013). Modified bacterial cellulose tubes for regeneration of damaged peripheral nerves. Arch. Med. Sci..

[20] Martínez Ávila H., Schwarz S., Feldmann E.-M., Mantas A., von Bomhard A., Gatenholm P., Rotter N. (2014). Biocompatibility evaluation of densified bacterial nanocellulose hydrogel as an implant material for auricular cartilage regeneration. Appl. Microbiol. Biotechnol..

[21] Baah-Dwomoh A., Rolong A., Gatenholm P., Davalos R.V. (2015). The feasibility of using irreversible electroporation to introduce pores in bacterial cellulose scaffolds for tissue engineering. Appl. Microbiol. Biotechnol..

[22] Scherner M., Reutter S., Klemm D., Sterner-Kock A., Guschlbauer M., Richter T., Langebartels G., Madershahian N., Wahlers T., Wippermann J. (2014). In vivo application of tissue-engineered blood vessels of bacterial cellulose as small arterial substitutes: Proof of concept?. J. Surg. Res..

[23] Gatenholm P., Backdahl H., Tzavaras T.J., Davalos R.V., Sano M.B. (2012). Three-Dimensional Bioprinting of Biosynthetic Cellulose (BC) Implants and Scaffolds for Tissue Engineering. U.S. Patent.

[24] Laromaine A., Tronser T., Pini I., Parets S., Levkin P.A., Roig A. (2018). Free-standing three-dimensional hollow bacterial cellulose structures with controlled geometry: Via patterned superhydrophobic-hydrophilic surfaces. Soft Matter.

[25] Li Y., Jiang K., Feng J., Liu J., Huang R., Chen Z., Yang J., Dai Z., Chen Y., Wang N. (2017). Construction of Small-Diameter Vascular Graft by Shape-Memory and Self-Rolling Bacterial Cellulose Membrane. Adv. Healthc. Mater..

[26] Nagashima A., Tsuji T., Kondo T. (2016). A uniaxially oriented nanofibrous cellulose scaffold from pellicles produced by Gluconacetobacter xylinus in dissolved oxygen culture. Carbohydr. Polym..

[27] Seyama T., Suh E.Y., Kondo T. (2013). Three-dimensional culture of epidermal cells on ordered cellulose scaffolds. Biofabrication.

[28] An S.J., Lee S.H., Huh J.B., Jeong S.I., Park J.S., Gwon H.J., Kang E.S., Jeong C.M., Lim Y.M. (2017). Preparation and characterization of resorbable bacterial cellulose membranes treated by electron beam irradiation for guided bone regeneration. Int. J. Mol. Sci..

[29] Kumbhar J.V., Jadhav S.H., Bodas D.S., Barhanpurkar-Naik A., Wani M.R., Paknikar K.M., Rrajwade J.M. (2017). In vitro and in vivo studies of a novel bacterial cellulose-based acellular bilayer nanocomposite scaffold for the repair of osteochondral defects. Int. J. Nanomed..

[30] Yadav V., Sun L., Panilaitis B., Kaplan D.L. (2015). In vitro chondrogenesis with lysozyme susceptible bacterial cellulose as a scaffold. J. Tissue Eng. Regen. Med..

[31] Svensson A., Nicklasson E., Harrah T., Panilaitis B., Kaplan D.L., Brittberg M., Gatenholm P. (2005). Bacterial cellulose as a potential scaffold for tissue engineering of cartilage. Biomaterials.

[32] Andersson J., Stenhamre H., Bäckdahl H., Gatenholm P. (2010). Behavior of human chondrocytes in engineered porous bacterial cellulose scaffolds. J. Biomed. Mater. Res. Part A.

[33] Martínez Ávila H., Feldmann E.M., Pleumeekers M.M., Nimeskern L., Kuo W., de Jong W.C., Schwarz S., Müller R., Hendriks J., Rotter N. (2015). Novel bilayer bacterial nanocellulose scaffold supports neocartilage formation invitro and invivo. Biomaterials.

[34] Wang Y., Yuan X., Yu K., Meng H., Zheng Y., Peng J., Lu S., Liu X., Xie Y., Qiao K. (2018). Fabrication of nanofibrous microcarriers mimicking extracellular matrix for functional microtissue formation and cartilage regeneration. Biomaterials.

[35] Silva M.A., Leite Y.K.C., de Carvalho C.E.S., Feitosa M.L.T., Alves M.M.M., Carvalho F.A.A., Neto B.C.V., Miglino M.A., Jozala A.F., de Carvalho M.A.M. (2018). Behavior and biocompatibility of rabbit bone marrow mesenchymal stem cells with bacterial cellulose membrane. PeerJ.

[36] Czaja W.K., Young D.J., Kawecki M., Brown R.M. (2007). The future prospects of microbial cellulose in biomedical applications. Biomacromolecules.

[37] Chen S.-Q., Lopez-Sanchez P., Wang D., Mikkelsen D., Gidley M.J. (2018). Mechanical properties of bacterial cellulose synthesised by diverse strains of the genus Komagataeibacter. Food Hydrocoll..

[38] Benziman M., Haigler C.H., Brown R.M., White A.R., Cooper K.M. (1980). Cellulose biogenesis: Polymerization and crystallization are coupled processes in Acetobacter xylinum. Proc. Natl. Acad. Sci. USA.

[39] Zaar K. (1979). Visualization of pores (export sites) correlated with cellulose production in the envelope of the gram-negative bacterium Acetobacter xylinum. J. Cell Biol..

[40] Brown R.M., Willison J.H., Richardson C.L., Richardson C.L. (1976). Cellulose biosynthesis in Acetobacter xylinum: Visualization of the site of synthesis and direct measurement of the in vivo process. Proc. Natl. Acad. Sci. USA.

[41] Hu S.-Q., Gao Y.-G., Tajima K., Sunagawa N., Zhou Y., Kawano S., Fujiwara T., Yoda T., Shimura D., Satoh Y. (2010). Structure of bacterial cellulose synthase subunit D octamer with four inner passageways. Proc. Natl. Acad. Sci. USA.

[42] Hestrin S., Schramm M. (1954). Synthesis of cellulose by Acetobacter xylinum. II. Preparation of freeze-dried cells capable of polymerizing glucose to cellulose. Biochem. J..

[43] Bertani G. (2004). Lysogeny at mid-twentieth century: P1, P2, and other experimental systems. J. Bacteriol..

[44] Sun Q.-Y., Ding L.-W., He L.-L., Sun Y.-B., Shao J.-L., Luo M., Xu Z.-F. (2009). Culture of Escherichia coli in SOC medium improves the cloning efficiency of toxic protein genes. Anal. Biochem..

[45] Fang J., Kawano S., Tajima K., Kondo T. (2015). In Vivo Curdlan/Cellulose Bionanocomposite Synthesis by Genetically Modified *Gluconacetobacter xylinus*. Biomacromolecules.

[46] Khan S., Ul-Islam M., Ullah M.W., Ikram M., Subhan F., Kim Y., Jang J.H., Yoon S., Park J.K. (2015). Engineered regenerated bacterial cellulose scaffolds for application in in vitro tissue regeneration. RSC Adv..

[47] An J., Teoh J.E.M., Suntornnond R., Chua C.K. (2015). Design and 3D Printing of Scaffolds and Tissues. Engineering.

[48] Ludwicka K., Kolodziejczyk M., Gendaszewska-Darmach E., Chrzanowski M., Jedrzejczak-Krzepkowska M., Rytczak P., Bielecki S. (2018). Stable composite of bacterial nanocellulose and perforated polypropylene mesh for biomedical applications. J. Biomed. Mater. Res. Part B Appl. Biomater..

[49] Gerding M.A., Ogata Y., Pecora N.D., Niki H., De Boer P.A.J. (2007). The trans-envelope Tol-Pal complex is part of the cell division machinery and required for proper outer-membrane invagination during cell constriction in *E. coli*. Mol. Microbiol..

[50] Cascales E., Lloubès R., Sturgis J.N. (2001). The TolQ-TolR proteins energize TolA and share homologies with the flagellar motor proteins MotA-MotB. Mol. Microbiol..

[51] Inoue T., Shingaki R., Hirose S., Waki K., Mori H., Fukui K. (2007). Genome-wide screening of genes required for swarming motility in Escherichia coli K-12. J. Bacteriol..

[52] Ha D.-G., Kuchma S.L., O’Toole G.A. (2014). Plate-Based Assay for Swarming Motility in Pseudomonas aeruginosa. Methods Mol. Biol..

[53] Deditius J.A., Felgner S., Spöring I., Kühne C., Frahm M., Rohde M., Weiß S., Erhardt M. (2015). Characterization of Novel Factors Involved in Swimming and Swarming Motility in Salmonella enterica Serovar Typhimurium. PLoS ONE.

[54] Keshk S.M. (2014). Bacterial Cellulose Production and its Industrial Applications. J. Bioprocess. Biotech..

[55] Hesse S., Kondo T. (2005). Behavior of cellulose production of Acetobacter xylinum in13C-enriched cultivation media including movements on nematic ordered cellulose templates. Carbohydr. Polym..

[56] Kondo T., Kasai W., Nojiri M., Hishikawa Y., Togawa E., Romanovicz D., Brown R.M. (2012). Regulated patterns of bacterial movements based on their secreted cellulose nanofibers interacting interfacially with ordered chitin templates. J. Biosci. Bioeng..

[57] Phan A.D.T., Netzel G., Wang D., Flanagan B.M., D’Arcy B.R., Gidley M.J. (2015). Binding of dietary polyphenols to cellulose: Structural and nutritional aspects. Food Chem..

[58] Garg K., Ryan J.J., Bowlin G.L. (2011). Modulation of mast cell adhesion, proliferation, and cytokine secretion on electrospun bioresorbable vascular grafts. J. Biomed. Mater. Res. Part A.

[59] Theoharides T.C., Alysandratos K.D., Angelidou A., Delivanis D.A., Sismanopoulos N., Zhang B., Asadi S., Vasiadi M., Weng Z., Miniati A. (2012). Mast cells and inflammation. Biochim. Biophys. Acta Mol. Basis Dis..

[60] Urb M., Sheppard D.C. (2012). The role of mast cells in the defence against pathogens. PLoS Pathog..

