# Supplementary material for: Scaffolds for Chondrogenic Cells Cultivation Prepared from Bacterial Cellulose with Relaxed Fibers Structure Induced Genetically
Source: Nanomaterials (Basel). 2018 Dec 17;8(12):1066. doi: 10.3390/nano8121066 (PMC6315621; doi:10.3390/nano8121066)
Supplement: Supplementary file 1 [file nanomaterials-08-01066-s001.pdf]

Supplementary material

Table S1 Sequences of DNA oligonucleotides used in PCR

Primer	Sequence (5′-3′)
pTI-motAB_for	CGCGGATCCGTGACACGACCGACGACCTA
pTI_motAB_rev	CCCAAGCTTTCAGCGATCCGTCAGGCGGA
pTI_for	AAAATCTTCTCTCATCCG
pTI_rev	ACACAGGAAACAGACCAT

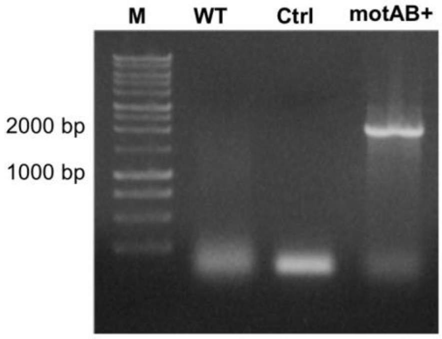

Figure S1 Confirmation of *K. hansenii* ATCC 23769 strain transformation by colony PCR with vector-specific primers (pTI_for, pTI_rev primers, Table S1). Empty vector (line Ctrl) was a source of product ~100 bp in length. Product of length ~2000 bp is of expected size for motAB insert cloned into pTI99A vector. Smear visible on the line WT and in the bottom of motAB+ line is due to unspecific amplification form genomic DNA. PCR was done without DNA isolation – directly on biomass from selective agar plate.

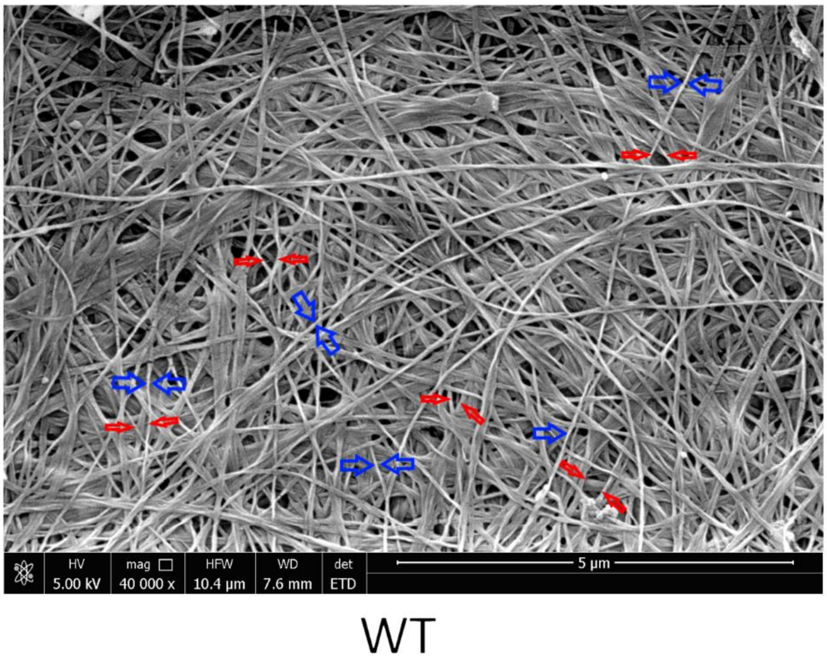

Figure S2. Example of fiber width and pore sizes estimation. By the blue arrows exemplary fibers widths are shown, by red arrows exemplary pores dimensions are shown.

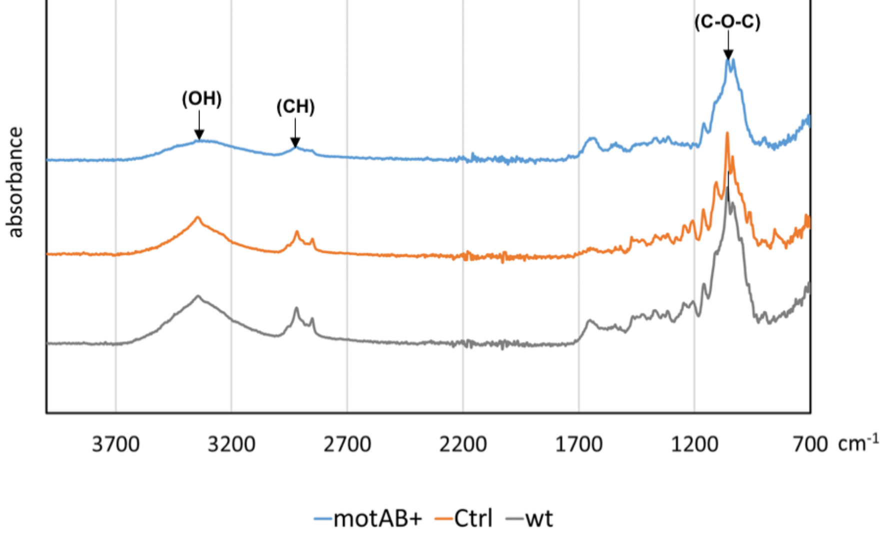

Figure S3. FTIR spectra collected for freeze-dried membranes produced by *K. hansenii* ATCC 23769 wild type strain and its variants: control (transformed with pTI99A vector) and mutant (motAB+ strain).

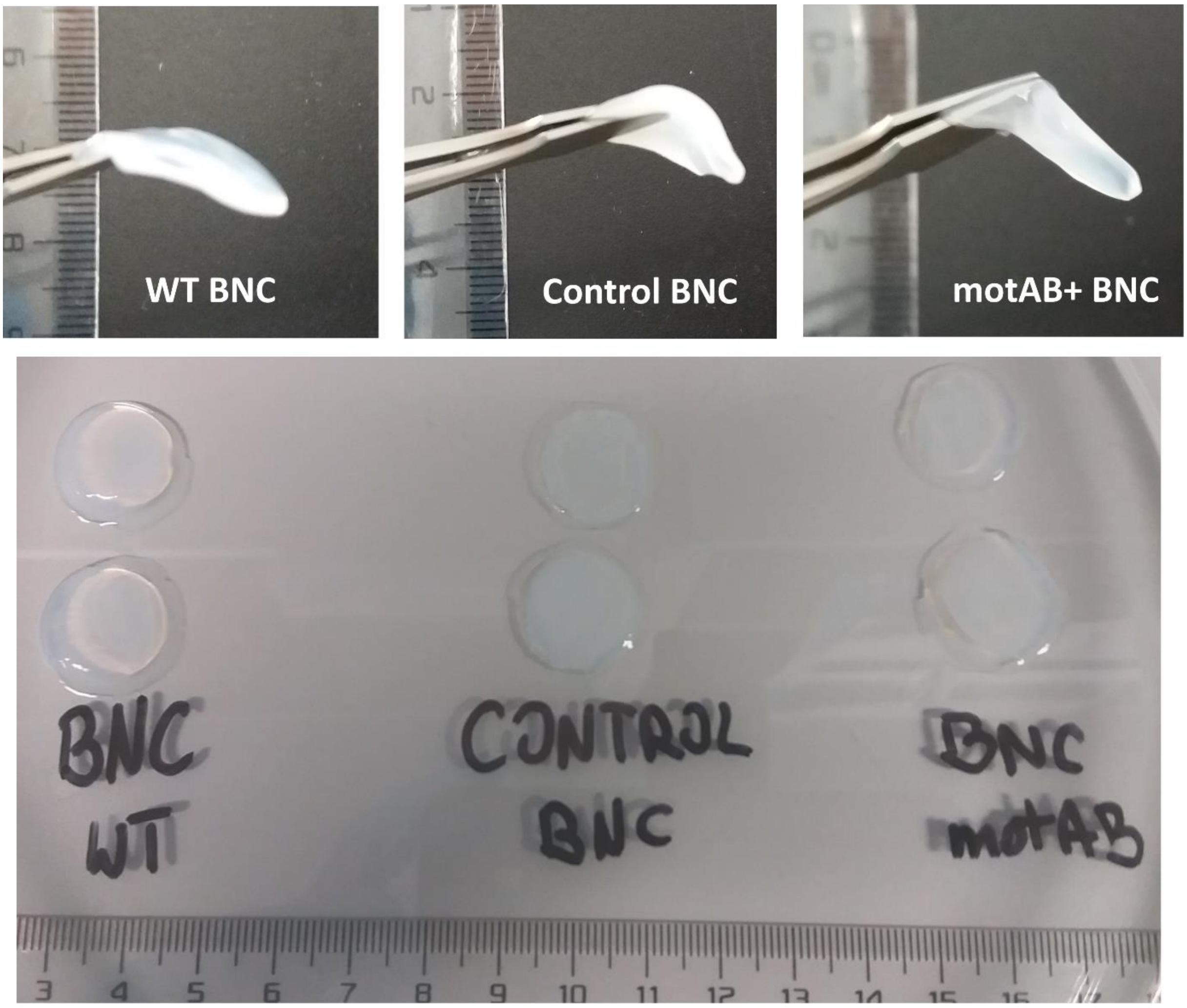

Figure S4. Macroscopic appearance of BNC scaffolds used in the study. Membranes shown were harvested from 24-well plates and washed. After sterilization in fresh 24-well plate they were used for cells seeding (WT BNC = cellulose produced by a wild type *K. hansenii* ATCC 23769 strain; Control BNC = cellulose produced by a *K. hansenii* ATCC 23769 strain transformed with pTI99 vector; BNC motAB+ = cellulose produced by a mutant strain)

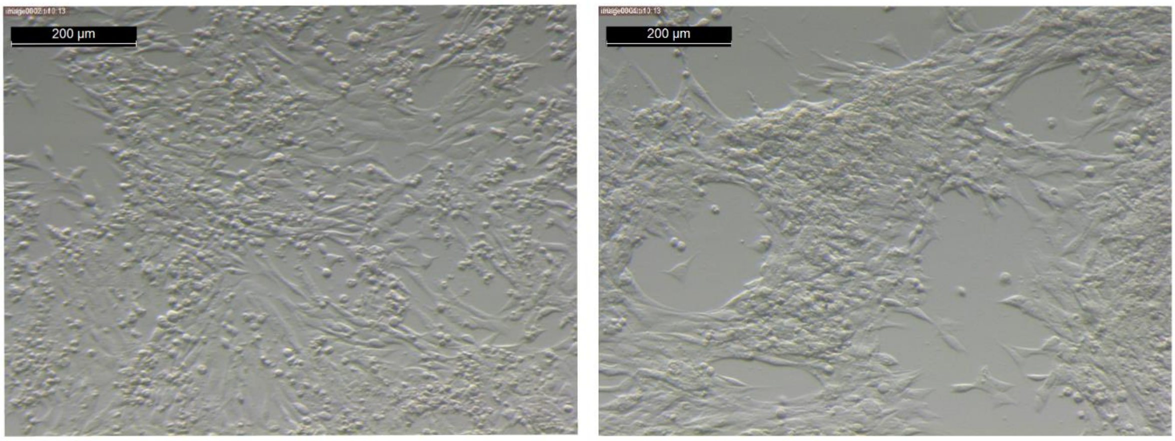

Plastic NDM Plastic DM

Figure S5. Morphology of ATDC5 cell line grown on 2D support was observed under light M205 microscope, equipped with a Leica MC170 HD camera. Representative images (100× magnification) from 3-week long culture are shown.

Plastic NDM – cells cultured in Non-Differentiating (growth) medium (content given in Materials and Methods of the main body of article);

Plastic DM – cells cultured in differentiation medium (obtained by combination of DMEM/F12 with Insulin-Transferrin-Selenium (ITS, Thermo Fisher Scientific, MA, USA), 100 nM dexamethasone (Sigma Aldrich, MO, USA), 10 ng/ml transforming growth factor – β (Sigma Aldrich, MO, USA), and 50 mg/mL ascorbic acid (Avantor Performance Materials, Poland)).

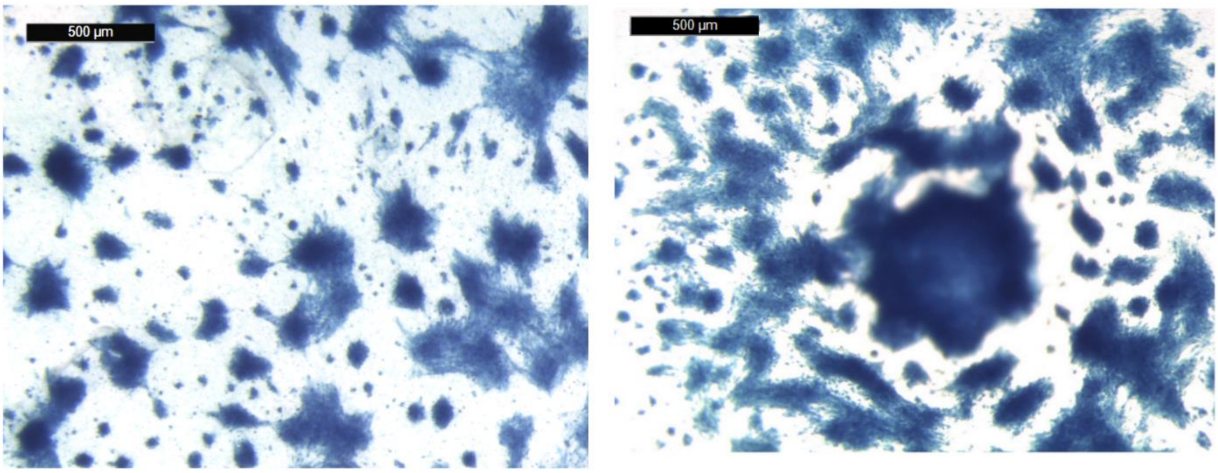

Control BNC motAB+ BNC

Figure S6. Morphology of ATDC5 cell line grown on BNC scaffolds, stained with Alcian blue, was observed under light M205 microscope, equipped with a Leica MC170 HD camera. Representative images (100× magnification) from 3-week long culture are shown.
